# Synthesis and Pharmacological Evaluation of Modified Adenosines Joined to Mono-Functional Platinum Moieties

**DOI:** 10.3390/molecules19079339

**Published:** 2014-07-03

**Authors:** Stefano D’Errico, Giorgia Oliviero, Nicola Borbone, Vincenzo Piccialli, Brunella Pinto, Francesca De Falco, Maria Chiara Maiuri, Rosa Carnuccio, Valeria Costantino, Fabrizia Nici, Gennaro Piccialli

**Affiliations:** 1 Dipartimento di Farmacia, Università degli Studi di Napoli “Federico II”, via D. Montesano, 49, 80131 Napoli, Italy; E-Mails: stefano.derrico@unina.it (S.D.); borbone@unina.it (N.B.); brunella.pinto87@gmail.com (B.P.); francesca.defalco@unina.it (F.D.F.); mariachiara.maiuri@unina.it (M.C.M.); rosa.carnuccio@unina.it (R.C.); valeria.costantino@unina.it (V.C.); fabrizia.nici@unina.it (F.N.); picciall@unina.it (G.P.); 2 Dipartimento di Scienze Chimiche, Università degli Studi di Napoli “Federico II”, via Cintia, 21, 80126 Napoli, Italy; E-Mail: vinpicci@unina.it; 3 INSERM U848, IGR, 39 rue C. Desmoulins, 94805 Villejuif, France

**Keywords:** platinum complexes, nucleosides, cisplatin, chemotherapy

## Abstract

The synthesis of four novel platinum complexes, bearing *N*^6^-(6-amino-hexyl)adenosine or a 1,6-di(adenosin-*N*^6^-yl)-hexane respectively, as ligands of mono-functional cisplatin or monochloro(ethylendiamine)platinum(II), is reported. The chemistry exploits the high affinity of the charged platinum centres towards the N7 position of the adenosine base system and a primary amine of an alkyl chain installed on the C6 position of the purine. The cytotoxic behaviour of the synthesized complexes has been studied in A549 adenocarcinomic human alveolar basal epithelial and MCF7 human breast adenocarcinomic cancer cell lines, in order to investigate their effects on cell viability and proliferation.

## 1. Introduction

The discovery of the anti-proliferative properties of cisplatin [1] marked the beginning of modern chemotherapy based on the use of metal complexes capable of blocking the replication of cancer cells targeting the nuclear DNA [2,3]. In fact, it is widely accepted that cisplatin, once inside the cell, may form a highly reactive species [4,5] that can react with DNA through the formation of intra-strand linkages [6,7]. Such linkages alter the secondary structure of DNA, resulting in an inhibition of transcription and replication, ultimately leading to cell death [4]. However, the poor solubility in biological fluids [8], the serious side effects [9,10], and, more importantly, the intrinsic and acquired resistance of many types of tumors [5], have limited its use in the clinic. Carboplatin and oxaliplatin, second and third generation anti-neoplastic agents, respectively, are able to enhance the quality of life of patients in terms of dosage and drug administration. However, they can still trigger mechanisms of resistance (intrinsic and/or acquired) during repeated cycles of oncological treatment [5]. It was then discovered that the presence of more charged metal centres separated by unbranched alkylamine chains leads to complexes capable of being uptaken by cells via active import [11] and of overcoming the intrinsic and/or acquired resistance in some tumors [6,12,13,14,15]. Based on the understanding that they may exert anti-tumoral activities through inter-strand linkages with DNA that cannot be repaired by enzymes [16], many multinuclear platinum complexes have recently been synthesized, by varying the diamine backbone chain length, or by introducing modified linkers [8,10,11,12,17,18]. In addition, new Pt-based compounds have been obtained by conjugating biologically important substances or drugs to Pt-containing subunits [19,20,21,22,23,24] and novel drug-delivery based methodologies have been explored to vehiculate platinum-based anti-cancer complexes directly against tumors [25,26].

Nucleoside and nucleotide anti-metabolites and their base analogues are able to inhibit specific pathways of the cancer cell metabolism by blocking the biosynthesis or the function of nucleic acids. For example, the combination of cisplatin and 5-fluorouracil, a chemotherapeutic agent that inhibits thymidylate synthase, has been extensively used in clinical practice to treat various types of cancer. Acyclovir, a guanosine nucleoside analogue containing an open-chain sugar surrogate, has been used as a ligand for platinum and the corresponding complex exhibits high *in vitro* activity against various *herpes* viruses [27]. Furthermore, the synthesis and the preliminary pharmacological activity of novel modified adenosines and thymidines, employed as N-donor ligands of platinum(II) dichloride complexes, have been recently reported [28,29].

**Figure 1:**
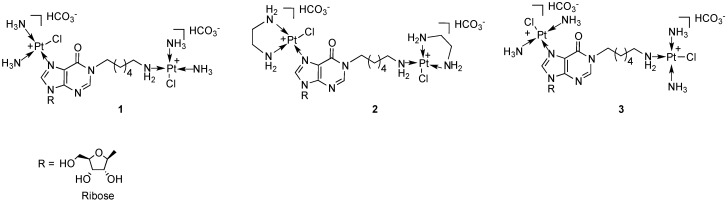
Bis-platinated nucleoside complexes synthesized starting from inosine [30].

Recently, we have reported the solid-phase synthesis and the pharmacological activity of the first examples of bis-platinated nucleoside complexes in which the mono-functional metal is linked both to N-7 of the purine nucleus of inosine and to the terminal amino-group of a hexylamine side chain installed on N-1 (compounds **1**–**3**, Figure 1) [30]. The amino-alkyl chain was introduced on the purine base system through a chemical strategy recently developed by us [30,31,32,33,34,35,36]. These complexes showed very good water solubility thanks to the charged platinum centres and to the ribose hydroxyl groups. They were tested against four different human tumor cell lines and, in particular, the complex bearing two monofunctional cisplatin units was revealed to be more cytotoxic than cisplatin against the MCF7 cancer cell line in a short-term exposure assay [30]. 

**Scheme 1:**
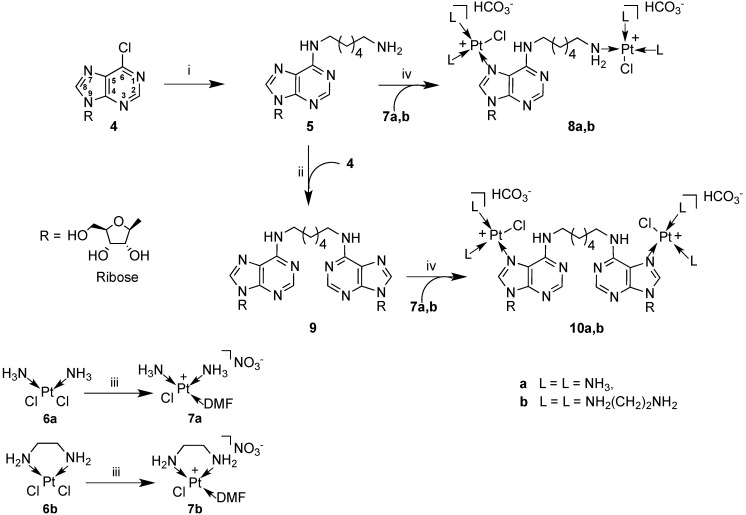
Synthesis of complexes **8** and **10**.

In the light of the above results, to investigate the importance of nucleoside scaffolds in the construction of novel platinum complexes we have speculated about the antitumoral activity of cisplatin-adenosine complexes. As a result, in this paper we report on the synthesis of four dinuclear platinum complexes **8a**,**b** and **10a**,**b** (Scheme 1) carrying *N*^6^-(6-aminohexyl)adenosine (compound **5**, Scheme 1) or a 1,6-di-(adenosin-*N*^6^-yl)-hexane (compound **9**, Scheme 1) respectively, as ligands of mono-functional cisplatin or monochloro(ethylene diamine)platinum(II) (compounds **7a** and **7b**, respectively, Scheme 1).

*Reagents and conditions*: (i) NH_2_(CH_2_)_6_NH_2_ (7 equiv.), TEA, EtOH, 3 h, reflux; (ii) **4** (1.5 equiv.), TEA, EtOH, 5 h, reflux; (iii) AgNO_3_ (0.9 equiv.), DMF, 16 h, r.t.; (iv) **7a**,**b**, 16 h, r.t..

In the case of complexes **8a**,**b** the monofunctional platinum centres are linked to the N-7 of the purine base and to the terminal amino group of a hexylamine side chain bonded to N-6, whereas for the complexes **10a**,**b** they are both linked to N-7.

Platinum complexes, endowed with particular structural strains, have been designed to evaluate alternative interactions with DNA strands [8,12,17,37,38]. The novel prepared complexes **8a**,**b** and **10a**,**b** are characterized by a rigid planar purine substructure joined to a flexible hexylamine chain. On this basis, it could be expected that they may alter the binding mode with DNA through, for example, base-stacking or by the formation of multiple hydrogen bonds. Therefore, we have investigated the preliminary effects of these compounds on viability and proliferation in two different human cancer cell lines.

## 2. Results and Discussion

### 2.1. Synthesis and Characterization

The synthesis of the novel modified adenosine-based platinum complexes **8a**,**b** and **10a**,**b** is depicted in Scheme 1. 6-Cloropurine riboside **4** proved to be a good candidate to obtain the key intermediate **5** from which the construction of complexes **8a**,**b** and **10a**,**b** has been accomplished. As reported by Schammells *et al.* [39] seven equivalents of 1,6-diaminohexane have proved sufficient to convert **4** into **5** in a very good yield (86%), avoiding the formation of dimeric species, which would complicate the purification procedure. In fact, the recovery of **5** from the reaction mixture was easily performed thanks to its insolubility in EtOH upon cooling. Compound **9** was prepared by reacting **4** with a slight excess of **5**. During the reaction, this substance precipitated from the reaction mixture as a white solid which was then collected in 93% yield by filtration. This represents a very good improvement on the reported yield for **9** (12%) [40].

Next, the platinum-containing moieties were installed. In particular, treatment of **5** and **9** with a seven-fold excess of the suitable platinating complex **7a**,**b**, activated by overnight reaction with AgNO_3_ (0.9 equiv.) in DMF, furnished the bisplatinated compounds **8a**,**b** and **10a**,**b**. Purification of these substances could be accomplished by reverse-phase HPLC only using a gradient of CH_3_CN in 0.1 M triethylammonium bicarbonate buffer (TEAB) as the solvent mixture, whereas complex chromatographic patterns were observed in the absence of the TEAB.

The structures of complexes **8a**,**b** and **10a**,**b** (yields 58%–62%) were supported by 2D-NMR and positive mode high resolution mass spectrometry (HRMS) data; whereas their purity was ascertained by CHN analyses. 

**Figure 2:**
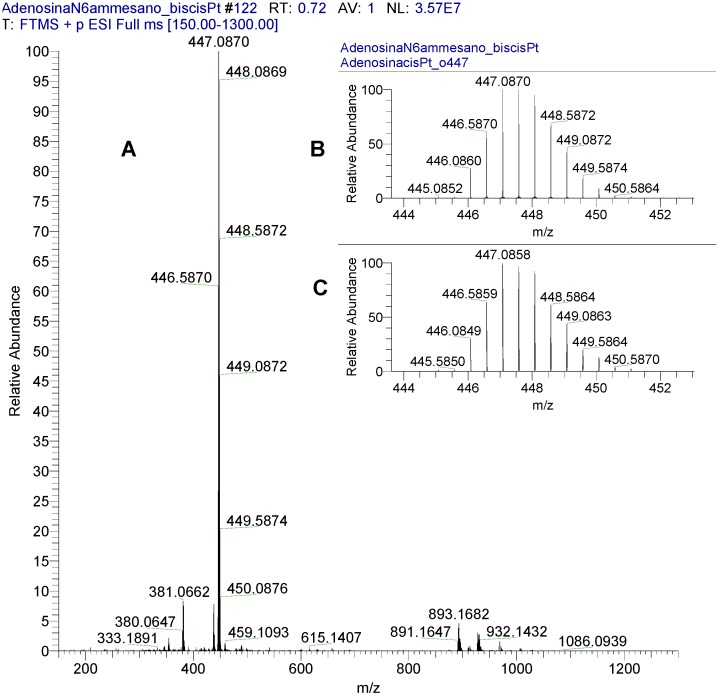
HRMS spectrum of complex **8a** (panel **A**). Expansion of the isotopic pattern of the base peak (panel **B**) and of the corresponding calculated one (panel **C**).

In Figure 2 (panel A) the representative HRMS spectrum of complex **8a** is reported; in particular, the isotopic pattern of the base peak (expansion, panel B) perfectly fits with that of the calculated one (expansion, panel C), confirming the presence of two Pt and two Cl atoms and a net 2+ charge.

In the^1^H-NMR spectra of **8a**,**b** and **10a**,**b** the downfield shift of H-8, compared with the resonance of the same proton in **5** and **7** (∆δ = 0.6 and 0.5, respectively), confirmed the presence of the N(7) → Pt bond in all these substances [41,42]. In Table 1 the differences between the ^13^C-NMR shifts of the Pt-coordinated and not-coordinated purine carbon atoms (∆δ = δ*_complex_* − δ*_ligand_*, ppm) are listed: in the case of complexes **8a**,**b** significant coordination shifts were found for the C-5 and C-8 atoms, whereas for the complexes **10a**,**b** the C-5 atoms underwent the major shifts. Further evidence of metallation at N-7 come from the increased acidity of the H-8 as indicated by the reduced intensity of the pertinent signal in the ^1^H-NMR spectra of **8a**,**b** and **10a**,**b**, when these complexes were dissolved in D_2_O and the spectra acquired after several hours [43]. ^1^H-NMR analyses excluded also the presence of equilibria involving migrations of the platinum moieties from N-7 to N-1 of the nucleobases; such migrations would shift the H-2 resonances of complexes **8a**,**b** and **10a**,**b** to higher frequences in comparison to the same signals of compounds **5** and **9**, respectively [44]. The resonances of the methylene protons belonging to the CH_2_-NH_2_-Pt moieties were seen as broad partly overlapped triplets in the range 2.3–2.7 ppm, in the proton spectra of **8b** and **10b**, likewise the protons of the methylene group geminal to platinum in **8a** resonated as a triplet at δ 2.6. In ^13^C-NMR spectra of **8a** and **8b** the 5 ppm downfield shift of the ω-methylene carbon could be a consequence of the NH_2_ platination.

In the UV spectra of the complexes **8a**,**b** and **10a**,**b** the maxima of absorption appeared red-shifted (∆λ = 10–13 nm) in respect to those of the free ligands, in accordance with literature data [45]. Comparison of the IR spectra of the ligands and complexes did not provide evidence about the bonding mode of the ligands in the complexes; in fact in the 4000–2700 cm^−1^ region very little information could be obtained about the nature of metal-ligand interaction, because of the presence of broad and intense bands belonging to hydrogen-bonded hydroxyls of the ribose moieties. The 1800–400 cm^−1^ region, where vibration frequencies of purine and ribose skeletons fall, did not furnish significant differences between platinated and not-platinated nucleosides. Furthermore, in the region 550–400 cm^−1^, where the resonances of Pt-N could be expected [45,46], no distinctive bands were observed.

**Table 1:** Differences between the ^13^C-NMR shifts of the Pt-coordinated and not-coordinated purine carbons (∆δ = δ*_complex_* − δ*_ligand_*, ppm).

Entry	^13^C-NMR (∆δ, ppm)				
	C-2	C-4	C-5	C-6	C-8
**8a** [39]	−1.0	−1.4	−2.9	0.9	2.1
**8b** [39]	−1.0	−1.3	−2.8	0.9	2.1
**10a**	−1.9	−2.3	−4.2	−0.1	0.9
**10b**	−2.0	−2.5	−4.2	0.1	1.1

### 2.2. Cytotoxicity Studies

**Figure 3:**
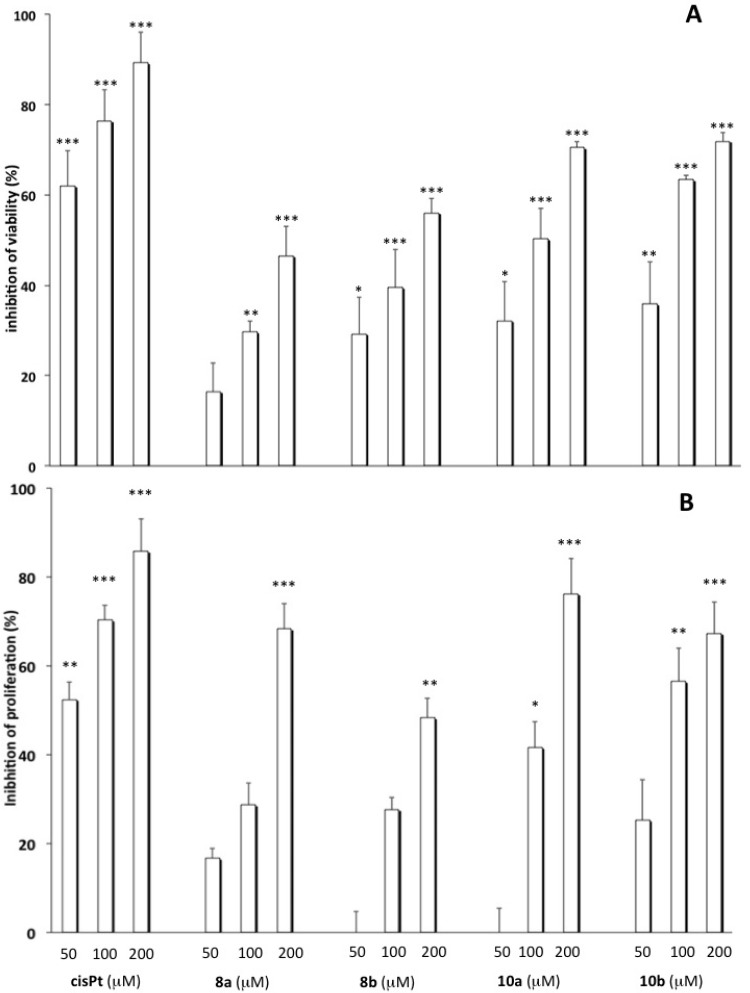
Effect of **8a**, **8b**, **10a** and **10b** on cell viability and proliferation. A549 cells were incubated with cisplatin, **8a**, **8b**, **10a** and **10b** (50, 100 and 200 µM) for 72 h. Thereafter, cell viability (panel **A**) and proliferation (panel **B**) were determined, respectively, by MTT and BrdU assay as described in the Experimental Section (*** *p* < 0.001, ** *p* < 0.01, * *p* < 0.05 *vs.* untreated cells).

The synthesized complexes were subjected to preliminary cell viability and proliferation assays. In particular, the cytotoxic behaviour was studied in A549 adenocarcinomic human alveolar basal epithelial and MCF7 human breast adenocarcinomic cancer cell lines by MTT and BrdU assays, to investigate the potential effects of platinum complexes on cell viability and proliferation, respectively. The incubation of A549 cells with **8a**,**b** and **10a,b** (50, 100 and 200 µM) for 72 h caused a concentration-dependent reduction of cell survival (Figure 3, panel A) as well as an inhibition of cell proliferation at the higher concentrations (Figure 3, panel B) compared to untreated cells. Indeed, comparable results were obtained when the cell number was directly determined by cell counting (data not shown). 

**Figure 4:**
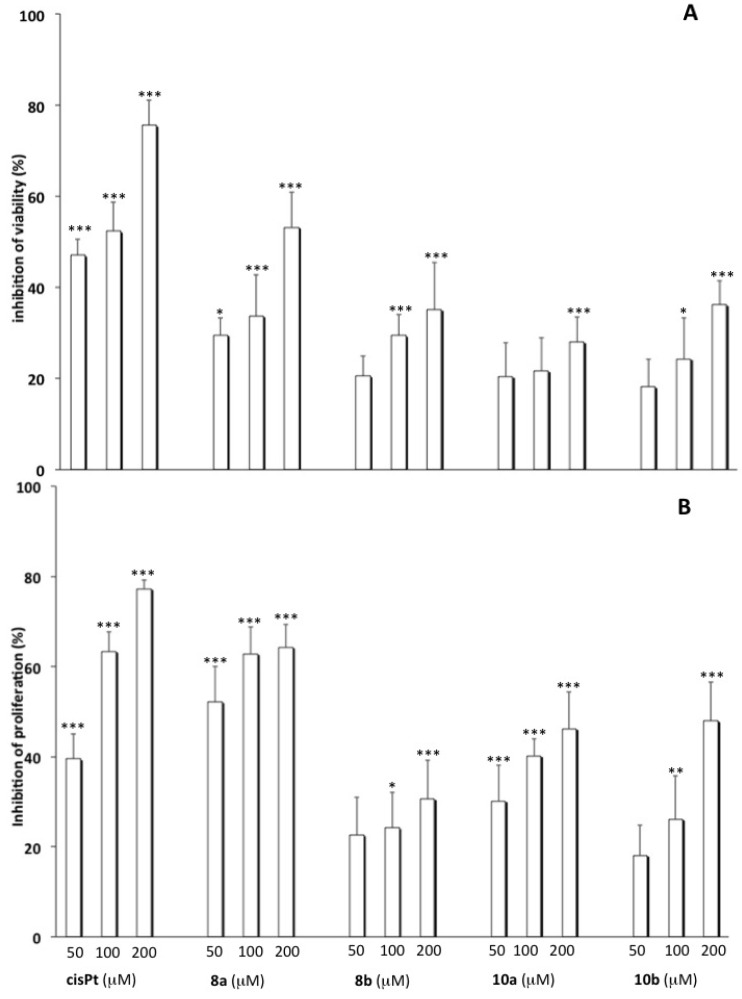
Effect of **8a**, **8b**, **10a** and **10b** on cell viability and proliferation. MCF7 cells were incubated with cisplatin, **8a**, **8b**, **10a** and **10b** (50, 100 and 200 µM) for 72 h. Thereafter, cell viability (panel **A**) and proliferation (panel **B**) were determined, respectively, by MTT and BrdU assay as described in the Experimental Section (*** *p* < 0.001, ** *p* < 0.01, * *p* < 0.05 *vs.* untreated cells).

The compounds **8b**, **10a** and **10b** (50, 100 and 200 µM) modified the viability and proliferation of MCF7 cells at higher concentrations, according to the published evidence that these cells have been associated with cisplatin resistance [47]. The compound **8a** proved to reduce the proliferation of the MCF7 cell line with a greater ability than the other compounds. Strikingly, compound **8a** was able to inhibit the cell proliferation slightly better than cisplatin, but only at 50 µM concentration (Figure 4).

## 3. Experimental Section

### 3.1. General Methods

All the reagents and solvents were obtained from commercial sources and used without further purification. ^1^H- and ^13^C-NMR spectra were acquired on Varian Mercury Plus 400 MHz instrument using D_2_O or (CD_3_)_2_SO as solvents. Chemical shifts were reported in parts per million (δ) relative to the residual solvent signal (^1^H: HDO 4.80; ^13^C: (CD_3_)(CD_2_H)SO 40.4) and assigned by 2D-NMR experiments. UV spectra were recorded on a Jasco V-530 UV spectrophotometer. IR spectra were recorded on a Jasco FT-IR 430 spectrophotometer. High-resolution MS spectra were recorded on a Thermo Orbitrap XL mass spectrometer using the electrospray ionization (ESI) technique in positive mode. Elemental analyses were performed on a Thermo Finnigan Flash EA 1112 CHN analyzer. RP-HPLC analyses and purifications were carried out on a Jasco UP-2075 Plus pump, equipped with a Jasco UV-2075 Plus UV detector, using a C-18 reverse-phase column (5 µm, 4.8 × 150 mm), eluted with a linear gradient of CH_3_CN in 0.1 M triethylammonium bicarbonate (TEAB) buffer (System A: from 0% to 50% in 90 min, flow 1.3 mL/min, or System B: from 0% to 100% in 90 min, flow 1.3 mL/min). Human alveolar basal carcinoma epithelial cells (A549) and human breast cancer cells (MCF7) were grown in Dulbecco’s modified Eagle’s medium (DMEM) supplemented with 10% Foetal bovine serum (FBS), 100 U/mL penicillin and 100 U/mL streptomycin at 37 °C under 5% CO_2_. The cell lines were all from the ATCC catalogue. All media and supplements for cell culture were purchased from Gibco-Invitrogen (GE Healthcare).

*N^6^-(6-Aminohexyl)adenosine* (**5**)*.* Compound **5** (326 mg) was obtained by reaction of **4** (297 mg, 01.04 mmol) in accordance to the procedure by Schammells *et al.* [37]. Spectroscopic data and yields were in agreement with those reported by the authors.

*1,6-Di-(adenosin-N^6^-yl)-hexane* (**9**)*.* Compound **5** (100 mg, 0.27 mmol), 6-chloropurine riboside (**4**, 116 mg, 0.40 mmol), Et_3_N (41 µL, 0.30 mmol) were refluxed in EtOH (1 mL). During the reaction, a colourless solid precipitated from the reaction. After 5 h it was filtered, washed with boiling EtOH and dried, giving **9** as a colourless powder (164 mg, 93%). El. An. Calcd. for C_26_H_36_N_10_O_8_: C, 50.64; H, 5.88; N, 22.72. Found: C, 50.68; H, 5.90; N, 22.69. ^1^H-NMR and UV data were in agreement with those reported in literature [38]. ^13^C-NMR (100 MHz, (CD_3_)_2_SO) δ 155.6 (C-2), 153.3 (C-6), 149.1 (C-4), 140.5 (C-8), 120.7 (C-5), 88.9 (C-1'), 86.9 (C-4'), 74.4 (C-2'), 71.6 (C-3'), 62.6 (C-5'), 40.8 (CH_2_NH, covered by residual solvent signal), 30.0 (CH_2_), 27.1 (CH_2_); IR (KBr pellet) 3336, 2935, 2861 1630, 1587, 1537, 1474, 1418, 1369, 1334, 1307, 1221, 1185, 1130, 1097, 1058, 985, 866, 822, 793, 641, 554 cm^−1^; *m/z* 639.2620 (HRESIMS) ([M + Na]^+^, C_26_H_36_N_10_NaO_8_, requires 639.2615).

### 3.2. General Procedure for the Preparation of Dinuclear Platinum Complexes **8a**,**b** and **10a**,**b**

In a representative experiment, cisplatin (57 mg, 0.19 mmol) was activated by treatment with AgNO_3_ (29.0 mg, 0.17 mmol) in DMF (2 mL) in the dark (16 h, r.t.). AgCl was removed by filtration and the resulting solution of [Pt(NH_3_)_2_(Cl)DMF]^+^(NO_3_)^−^ in DMF was added to compound **5** (10 mg, 0.027 mmol). The solution was shaken in the dark (16 h, r.t.) and then filtered over a GHP Acrodisc 13 mm syringe filter (0.45 µm GHP membrane). The filter was washed with the minimal amount of DMF and the clarified solution was then subjected to HPLC purification [system A for **8a**,**b** (t_R_: 38.7 min and 38.4 min respectively) and system B for **10a**,**b** (t_R_: 31.0 min and 31.5 min respectively), see General Methods]; the fractions containing the title compound were evaporated under reduced pressure and then lyophilized affording pure **8a**.

*Compound* **8a***.* Bis-bicarbonate salt (17 mg, 60%). El. An. Calcd. for C_18_H_40_Cl_2_N_10_O_10_Pt_2_: C, 21.24; H, 3.96; N, 13.76. Found: C, 21.21; H, 3.98; N, 13.79. ^1^H-NMR (400 MHz, D_2_O) δ 8.84 (s, 1H, H-8), 8.33 (s, 1H, H-2), 6.12 (bs, 1H, H-1'), 4.78–4.76 (m, 1H, H-2', partially covered by solvent signal), 4.43–4.36 (m, 1H, H-3'), 4.31–4.25 (m, 1H, H-4'), 3.97–3.76 (m, 2H, H_a,b_-5'), 3.67–3.55 (m, 2H, CH_2_N), 2.62 (t, *J* = 6.4 Hz, 1H, C*H*_2_NH_2_Pt), 1.86–1.72 (m, 2H, CH_2_), 1.71–1.59 (m, 2H, CH_2_), 1.57–1.34 (complex signal, 4H, 2 × CH_2_); ^13^C-NMR (100 MHz, D_2_O) δ 160.2 (2 × HCO_3_^−^), 153.7 (C-2), 153.2 (C-6), 146.8 (C-4), 141.6 (C-8), 116.8 (C-5), 89.1 (C-1'), 85.9 (C-4'), 73.9 (C-2'), 70.1 (C-3'), 61.0 (C-5'), 45.7 (CH_2_NH_2_), 40.7 (CH_2_NH), 29.8 (CH_2_), 28.0 (CH_2_), 25.4 (CH_2_), 25.2 (CH_2_). IR (KBr pellet) 3246, 2930, 1626, 1589, 1492, 1339, 1084, 1058, 835, 790, 553 cm^−1^; UV (H_2_O) λ_max_ = 277 nm; HRESI-MS: (*m*/*z*) 447.0870, calcd. [M]^2+^ 447.0859.

*Compound* **8b***.* Bis-bicarbonate salt (17 mg, 58%). El. An. Calcd. for C_22_H_44_Cl_2_N_10_O_10_Pt_2_: C, 24.70; H, 4.15; N, 13.09. Found: C, 24.73; H, 4.13; N, 13.06. ^1^H-NMR (400 MHz, D_2_O) δ 8.85 (s, 1H, H-8), 8.39 (s, 1H, H-2), 6.16 (d, *J* = 5.0 Hz, 1H, H-1'), 4.80 (m, 1H, H-2', covered by solvent signal), 4.48–4.41 (m, 1H, H-3'), 4.37–4.30 (m, 1H, H-4'), 4.00–3.84 (m, 2H, H_a,b_-5'), 3.78–3.67 (m, 2H, CH_2_N), 2.85–2.50 (complex signal, 10H, C*H*_2_NH_2_Pt and 4 × CH_2_ ethylene diamine moieties) 1.91–1.78 (m, 2H, CH_2_), 1.77–1.62 (m, 2H, CH_2_), 1.61–1.36 (complex signal, 4H, 2 × CH_2_); ^13^C-NMR (100 MHz, D_2_O) δ 160.2 (2 × HCO_3_^−^), 153.7 (C-2), 153.2 (C-6), 146.9 (C-4), 141.6 (C-8), 116.9 (C-5), 89.1 (C-1'), 85.9 (C-4'), 73.9 (C-2'), 70.1 (C-3'), 61.0 (C-5'), 48.3, 48.0, 47.1 (4 × CH_2_ ethylene diamine moieties), 45.9 (CH_2_NH_2_), 40.7 (CH_2_NH), 29.9 (CH_2_), 28.1 (CH_2_), 25.5 (CH_2_), 25.2 (CH_2_); IR (KBr pellet) 3406, 3208, 2931, 1627, 1589, 1470, 1336, 1303, 1232, 1079, 1054, 834, 790, 555, 472 cm^−1^; UV (H_2_O) λ_max_ = 278 nm; HRESI-MS: (*m*/*z*) 473.1037, calcd. [M]^2+^ 473.1031. 

*Compound* **10a***.* Bis-bicarbonate salt (12 mg, 60%). El. An. Calcd. for C_28_H_50_Cl_2_N_14_O_14_Pt_2_: C, 26.53; H, 3.97; N, 15.47. Found: C, 26.50; H, 3.95; N, 15.45. ^1^H-NMR (400 MHz, D_2_O) δ 8.85 (s, 2H, 2 × H-8), 8.26 (s, 2H, 2 × H-2), 6.14 (bs, 2H, 2 × H-1'), 4.80 (m, 2H, 2 × H-2', covered by solvent signal), 4.50–4.40 (m, 2H, 2 × H-3'), 4.38–4.29 (m, 2H, 2 × H-4'), 4.03–3.76 (complex signal, 6H, 2 × H_a,b_-5' and 2 × CH_a_N), 3.67–3.53 (m, 2H, 2 × CH_b_N), 1.94–1.75 (m, 4H, 2 × CH_2_), 1.73–1.51 (m, 4H, 2 × CH_2_); ^13^C-NMR (100 MHz, D_2_O) δ 160.2 (HCO_3_^−^), 153.7 (C-2), 153.2 (C-6), 146.8 (C-4), 141.4 (C-8), 116.5 (C-5), 89.1 (C-1'), 85.8 (C-4'), 74.0 (C-2'), 70.0 (C-3'), 61.0 (C-5'), 40.5 (CH_2_NH), 27.8 (CH_2_), 25.2 (CH_2_); IR (KBr pellet) 3280, 2933, 1626, 1589, 1492, 1415, 1339, 1306, 1230, 1085, 1059, 985, 864, 790, 552 cm^−1^; UV (H_2_O) λ_max_ = 277 nm; HRESI-MS: (*m*/*z*) 572.1213, calcd. [M]^2+^ 572.1226.

*Compound* **10b**. Bis-bicarbonate salt (13 mg, 62%). El. An. Calcd. for C_32_H_54_Cl_2_N_14_O_14_Pt_2_: C, 29.12; H, 4.12; N, 14.86. Found: C, 29.15; H, 4.10; N, 14.83. ^1^H-NMR (400 MHz, D_2_O) δ 8.81 (s, 2H, 2 × H-8), 8.24 (s, 2H, 2 × H-2), 6.14 (bs, 2H, 2 × H-1'), 4.80 (m, 2H, 2 × H-2', covered by solvent signal), 4.49–4.41 (m, 2H, 2 × H-3'), 4.37–4.31 (m, 2H, 2 × H-4'), 4.04–3.83 (m, 4H, 2 × H_a,b_-5'), 3.81–3.60 (m, 4H, 2 × CH_2_N), 2.84–2.50 (complex signal, 8H, 4 × CH_2_, ethylene diamine moieties), 1.95–1.74 (m, 4H, 2 × CH_2_), 1.72–1.52 (m, 4H, 2 × CH_2_); ^13^C-NMR (100 MHz, D_2_O) δ 160.1 (HCO_3_^−^), 153.6 (C-2), 153.2 (C-6), 146.6 (C-4), 141.6 (C-8), 116.5 (C-5), 89.1 (C-1'), 85.7 (C-4'), 74.1 (C-2'), 70.1 (C-3'), 61.2 (C-5'), 48.1, 47.3 (2 × CH_2_ ethylene diamine moieties) 40.5 (CH_2_NH), 27.7 (CH_2_), 25.1 (CH_2_); IR (KBr pellet) 3284, 2930, 1625, 1592, 1490, 1417, 1338, 1310, 1233, 1083, 1061, 980, 860, 791, 554 cm^−1^; UV (H_2_O) λ_max_ 276 nm; HRESI-MS: (*m*/*z*) 598.1391, calcd. [M]^2+^ 598.1382.

### 3.3. MTT Viability Assay

The cells were plated in 96 culture wells (20 × 10^4^ cells/well) and allowed to adhere overnight. Thereafter, the medium was replaced with fresh medium and the cells were incubated in the absence or presence of cisplatin, **8a**, **8b**, **10a** and **10b** (50, 100 and 200 µM). After 72 h, the cell viability was determined by using 3-(4,5-dimethylthiazol-2yl)-2,5-diphenyl-2H-tetrazoliumbromide (MTT) conversion assay [48]. Briefly, 10 µL of MTT (5 mg/mL) were added to the cells and incubated for an additional 3 h. After this time point, the cells were lysed and the dark blue crystals solubilized with 150 µL of a solution containing 50% (*v:v*) DMF, 20% (*w:v*) SDS with an adjusted pH of 4.5. The optical density (OD) of each well was measured with a microplate spectrophotometer (Multiskan MCCC/340, Titertek, Huntsville, AL, USA) equipped with a 620 nm filter. The experiment was performed twice in triplicate. The vitality inhibition, induced by each compound at the indicated concentrations, was expressed as a percentage *versus* the untreated cells (the control). The viable cells were also counted by the trypan blue exclusion assay and light microscopy.

### 3.4. BrdU Cell Proliferation Assay

The cells were plated onto 96-well plates (2 × 10^4^ cells/well) overnight. Then, the medium was replaced with fresh medium and the cells were incubated in the absence or presence of cisplatin (50, 100 and 200 µM), **8a**, **8b**, **10a** and **10b** (50, 100 and 200 µM). After 72 h, 5-bromo-2'-deoxyuridine (BrdU; 10 µM) was added and the cells were cultured for a further 12 h. The mitogenic activity was determined according to the manufacturer’s instructions (BrdU cell proliferation assay kit, Cell Signaling). The experiment was performed three times in triplicate. The proliferation inhibition, induced by each compound at the indicated concentrations, was expressed as a percentage *versus* the untreated cells (the control). 

### 3.5. Statistical Analysis

The results are expressed as the means ± SEM of n experiments. The statistical significance was calculated by one-way analysis of variance (ANOVA) and Bonferroni-corrected *p*-value for multiple comparison testing. The level of statistically significant difference was defined as *p* < 0.05.

## 4. Conclusions

In summary, in this paper we have reported the synthesis of four novel platinum complexes embodying a *N*^6^-(6-aminohexyl)adenosine or a 1,6-di-(adenosin-*N*^6^-yl)-hexane as ligands of monofunctional cisplatin or monochloro(ethylene diamine)platinum(II) moieties, starting from commercially available 6-chloropurine riboside. The designed synthetic route allowed us to obtain the desired platinum complexes without blocking the ribose hydroxyl groups and through easy purification steps.

The effects of these platinum complexes on cell viability and proliferation have been studied in A549 and MCF7 cell lines. Our data demonstrate that these compounds are able to inhibit the survival and proliferation of sensitive cancer cells. Nevertheless, cisplatin remains the most active molecule under our experimental conditions. Further experiments are necessary to understand the mechanism by which **8a** penetrates into the cells and inhibits cell proliferation in the MCF7 cell line. 
